# Suppression Analysis of *esa1* Mutants in *Saccharomyces cerevisiae* Links *NAB3* to Transcriptional Silencing and Nucleolar Functions

**DOI:** 10.1534/g3.112.003558

**Published:** 2012-10-01

**Authors:** Christie S. Chang, Astrid Clarke, Lorraine Pillus

**Affiliations:** Division of Biological Sciences, University of California–San Diego, La Jolla, California 92093-0347; Moores Cancer Center, University of California–San Diego, La Jolla, California 92093-0347

**Keywords:** Nrd1, Sen1, chromatin, nonhistone acetylation, KAT

## Abstract

The acetyltransferase Esa1 is essential in the yeast *Saccharomyces cerevisiae* and plays a critical role in multiple cellular processes. The most well-defined targets for Esa1 are lysine residues on histones. However, an increasing number of nonhistone proteins have recently been identified as substrates of Esa1. In this study, four genes (*LYS20*, *LEU2*, *VAP1*, and *NAB3*) were identified in a genetic screen as high-copy suppressors of the conditional temperature-sensitive lethality of an *esa1* mutant. When expressed from a high-copy plasmid, each of these suppressors rescued the temperature-sensitivity of an *esa1* mutant. Only *NAB3* overexpression also rescued the rDNA-silencing defects of an *esa1* mutant. Strengthening the connections between *NAB3* and *ESA1*, mutants of *nab3* displayed several phenotypes similar to those of *esa1* mutants, including increased sensitivity to the topoisomerase I inhibitor camptothecin and defects in rDNA silencing and cell-cycle progression. In addition, nuclear localization of Nab3 was altered in the *esa1* mutant. Finally, posttranslational acetylation of Nab3 was detected *in vivo* and found to be influenced by *ESA1*.

Nucleosomes containing the core histones (H2A, H2B, H3, and H4) form the basic packaging unit of DNA that organizes chromatin into higher-order structures. The N-terminal tails of histones are subject to multiple covalent modifications that can influence gene expression locally at specific promoters or within large regions of chromatin. Increased histone acetylation is associated with both transcriptional activation and repression. Lysine acetyltransferases (KAT), the enzymes that catalyze the acetylation reaction on histones, have been ascribed multiple cellular functions. Recently, nonhistone targets have also been identified for many KATs, including Esa1 (Lin *et al.* 2009) [reviewed in Yang and Seto (2008)].

The Esa1 KAT of *Saccharomyces cerevisiae* is a member of the deeply conserved MYST family of acetyltransferases and is essential in yeast (Smith *et al.* 1998a; Clarke *et al.* 1999). Esa1 is the catalytic component of the NuA4 and piccolo complexes that acetylate histone H4, H2A, and its variant H2A.Z (Allard *et al.* 1999; Babiarz *et al.* 2006; Keogh *et al.* 2006; Millar *et al.* 2006). Many of the NuA4 subunits, including Esa1, are essential (Galarneau *et al.* 2000; Loewith *et al.* 2000; Eisen *et al.* 2001), indicating that this complex has critical cellular roles.

Esa1 has a role in regulating expression of ribosomal protein genes (Reid *et al.* 2000). Further, genome-wide expression analysis reveals widespread transcriptional changes in *esa1* mutants (Durant and Pugh 2006), and genome-wide binding profiles show Esa1 bound to the promoters of actively transcribed genes (Robert *et al.* 2004). Esa1 also functions in transcriptional silencing of the rDNA and at telomeres (Clarke *et al.* 2006). The variety of genomic targets identified thus far suggests Esa1 activity regulates transcription at many loci, indicative of its function in multiple cellular processes.

Genetic analysis further defines Esa1’s role in diverse cellular functions. Temperature-sensitive mutants of *esa1* display a G2/M cell-cycle arrest at the restrictive temperature that is dependent upon the *RAD9* DNA damage checkpoint (Clarke *et al.* 1999) and are hypersensitive to the topoisomerase I inhibitor camptothecin (Bird *et al.* 2002). Esa1 localizes to double-strand breaks where it functions in repair of DNA damage (Downs *et al.* 2004). Together, these results suggest Esa1 activity is required for cell-cycle regulation and genomic integrity, although Esa1’s catalytic activity may not be its only essential role (Decker *et al.* 2008).

Suppression analyses have linked *ESA1* to the deacetylase Sir2, a key silencing protein. Overexpression of Sir2 was found to suppress *esa1* rDNA-silencing defects, thereby suggesting that Sir2 and Esa1 may function coordinately to silence the rDNA array (Clarke *et al.* 2006). Several other studies have identified additional suppressors of conditional alleles of *esa1* (Biswas *et al.* 2008; Lin *et al.* 2008; Chang and Pillus 2009; Scott and Pillus 2010).

To pursue genetic interactors of *ESA1*, a dosage suppression screen was performed on an *esa1* mutant. Of the four high-copy suppressors identified, *NAB3* became a focus for two primary reasons. First, only *NAB3* overexpression rescued both the temperature-sensitivity and the silencing defects of *esa1* mutants. Second, *NAB3* has known roles in RNA processing, and this functional connection to Esa1 may establish a novel link between two nuclear processes. Numerous studies have characterized roles for Nab3 and its binding partner Nrd1 in 3′-end processing of several classes of small noncoding RNAs [reviewed in Lykke-Andersen and Jensen (2007)]. These classes of RNAs include small-nuclear (sn) RNAs, small-nucleolar (sno) RNAs, and cryptic unstable transcripts (CUT). Nab3 and Nrd1 each recognize specific RNA sequences for 3′-end formation and transcription termination (Carroll *et al.* 2004).

This study reports new mutant phenotypes of *nab3*, revealing roles for Nab3 in rDNA silencing, the DNA damage response, and cell-cycle progression. Further, Nab3 was found to be posttranslationally modified by acetylation. This acetylation was reduced in an *esa1* conditional mutant that displays reduced Esa1 acetyltransferase activity, providing evidence that Nab3 is a nonhistone substrate of Esa1 whose function may be influenced by acetylation.

## Materials and Methods

### Dosage suppressor screen

A *URA3*-marked 2μ genomic library (generously provided by P. Hieter) was transformed into two isolates of the *esa1-414* strain LPY3291 in six independent experiments, yielding a total of 130,000 transformants with an approximate 70-fold coverage of the genome. Transformants were grown under permissive conditions on SC-Trp-Ura plates, and then replica-plated and incubated at 28°, 35°, and 37°. Two hundred colonies were able to grow at 35° but not 37° (this was a secondary screen used to avoid recovering wild-type *ESA1*). These candidates were tested for plasmid dependence by growing original transformants on 5-fluoroorotic acid (5-FOA) plates. The resulting resistant strains, which had lost the *URA3*-marked plasmid, were tested for temperature sensitivity at 35°. This resulted in 34 suppressor strains being classified as plasmid-dependent. Suppressing plasmids were rescued from yeast, and inserts were sequenced using T3 and T7 primers. Of the 34 plasmids, 22 were WT *ESA1*, 3 were unidentified, and the remaining 9 comprised six independent clones containing one of the four following genes: *LYS20*, *NAB3*, *VAP1*, or *LEU2*. Library fragments that contained multiple ORFs were dissected by subcloning to identify the gene responsible for suppression. Strategy for identification of the four suppressors is described in detail (Clarke 2001). The plasmid subclones were retransformed into LPY3291 to confirm the suppressing phenotype.

### Yeast methods and strain and plasmid construction

All yeast strains and plasmids used in this study are listed in Tables 1 and 2. The silencing markers rDNA::*ADE2-CAN1* (Fritze *et al.* 1997) and TELVR::*URA3* (Renauld *et al.* 1993) were introduced through standard genetic crosses. All *nab3-10* strains originate from YPN100 (provided by M. Swanson) (Conrad *et al.* 2000). Nab3 Flag-tagging was carried out by amplification of pFA6a-2FLAG-*kanMX6* and transformation into LPY5 (W303-1a) using the method described (Longtine *et al.* 1998) to make LPY15000. All library plasmids are in the pRS202 (pLP1402) backbone. pLP1238 (*NAB3* in pRS202) and pLP2018 (*NAB3* in pRS426) were subcloned from pLP1419 (*NAB3* library construct) using *Eco*RI and *Xho*I. pLP1310 (*NAB3* in pLP271) was subcloned from pLP1419 using *Eco*RI. Dilution assays for growth, silencing, and drug sensitivity were performed as described (Chang and Pillus 2009) and represent 5-fold serial dilutions starting from an A_600_ of 1.0. Images were captured after 2–4 days of growth at the indicated temperatures.

**Table 1:** Yeast strains used in this study

Strain	Genotype	Reference
LPY5 (W303-1a)	*MAT*a *ade2-1 can1-100 his3-11,15 leu2-3,112 trp1-1 ura3-1*	Thomas and Rothstein 1989
LPY3291	*MAT*a *his3Δ200 leu2-3,112 trp1Δ1 ura3-52 esa1Δ*::*HIS3* + pLP863 (*esa1-414*)	Clarke *et al.* 1999
LPY4774	W303 *MAT*a *esa1-414*	
LPY4909	W303 *MAT*α rDNA::*ADE2-CAN1*	Clarke *et al.* 2006
LPY4911	W303 *MAT*α *esa1-414* rDNA::*ADE2-CAN1*	Clarke *et al.* 2006
LPY4917	W303 *MAT*α TELVR::*URA3*	Clarke *et al.* 2006
LPY4919	W303 *MAT*α *esa1-414* TELVR::*URA3*	Clarke *et al.* 2006
LPY4979	W303 *MAT*α *sir2Δ*::*HIS3* TELVR::*URA3*	
LPY5406	W303 *MAT*a *nab3-10* rDNA::*ADE2-CAN1*	
LPY5407	W303 *MAT*a *nab3-10* TELVR::*URA3*	
LPY10622	W303 *MAT*a *nab3-10*	
LPY11286	W303 *MAT*a *nab3-10 adh4*::*ADE2* TELVIIL	
LPY11300	W303 *MAT*a *adh4*::*ADE2* TELVIIL	
LPY12154	W303 *MAT*a *rpd3*::*kanMX*	Chang and Pillus 2009
LPY15000	W303 *MAT*a *NAB3-2Flag*::*kanMX*	
LPY15004	W303 *MAT*a *esa1-414 NAB3-2Flag*::*kanMX*	

Except where noted, strains were constructed during the course of this study or are part of the standard lab collection.

**Table 2:** Plasmids used in this study

Plasmid (Alias)	Description	Source/Reference
pLP362 (pRS426)	Vector *URA3* 2µ	Sikorski and Hieter 1989
pLP1402 (pRS202)	Library vector *URA3* 2μ	P. Hieter
pLP37	*SIR2 URA3* 2μ	
pLP271	Vector *TRP1* 2µ	
pLP796	*ESA1 URA3* 2μ	Clarke *et al.* 2006
pLP798	*ESA1 TRP1* 2µ	
pLP863	*esa1-414 TRP1* CEN	Clarke *et al.* 1999
pLP1238	*NAB3 URA3* 2μ	
pLP1259	*VAP1 URA3* 2μ	
pLP1310	*NAB3 TRP1* 2µ	
pLP1412	*LYS20 URA3* 2μ	
pLP1405	*LYS20*-library clone *URA3* 2μ	
pLP1406	*VAP1*-library clone *URA3* 2μ	
pLP1417	*LEU2*-library clone *URA3* 2μ	
pLP1419	*NAB3*-library clone *URA3* 2μ	
pLP2018	*NAB3 URA3* 2µ	
pLP2054	*NRD1 URA3* 2µ	

Except where noted, plasmids were constructed during the course of this study or are part of the standard lab collection. “Library clone” represents a clone obtained directly in the suppressor screen, whereas others are subclones as detailed in Clarke (2001).

### Northern analysis, protein immunoblots, and immunoprecipitations

RNA was isolated using the hot acid phenol protocol as described (Collart and Oliviero 2001). Northern blotting was performed as described (Cox and Walter 1996), and results were obtained by phosphorimager (Storm, GE Healthcare). Yeast extracts were prepared by bead beating as described previously (Clarke *et al.* 1999), separated on SDS-polyacrylamide gels (18% for detection of histones, 8% for Sir2 and Rpd3), and transferred to nitrocellulose (0.2 μm). Primary antisera used were anti-H4K5Ac (Serotec), anti-H4K8Ac (Serotec), anti-H4K12Ac (Serotec), anti-H4K16Ac (Upstate), anti-Sir2 (Garcia and Pillus 2002), anti-Rpd3 (Rundlett *et al.* 1996), anti-PGK (Baum *et al.* 1978), anti-FLAG (Sigma-Aldrich, F3165), and anti-acetyl-lysine (Cell Signaling, #9681). Secondary antibodies conjugated to horseradish peroxidase in combination with chemiluminescence reagents were used for detection on film. FLAG-Nab3 was immunoprecipitated with anti-FLAG M2 Affinity Gel (Sigma-Aldrich, A2220), eluted in SDS sample buffer, separated on a SDS-polyacrylamide gel, and immunoblotted with either anti-FLAG or anti-acetyl lysine. All experiments were performed in triplicate or more and a representative blot was chosen for quantification. Quantification of all immunoblots was performed with ImageQuant software.

### Nab3 and Sir2 immunofluorescence

Immunofluorescence was performed as described (Gotta *et al.* 1997; Stone *et al.* 2000). WT and *esa1* strains were grown in YPD for four hours at either 28° or 37°. Cells were fixed by adding paraformaldehyde to the cultures at a final concentration of 3.3% at 30° for 10 min. Samples were washed twice in YPD, resuspended at 1 ml per 0.1 g of cells in 0.1 M EDTA, KOH pH 8.0, and 19 mM DTT, and then incubated at 30° for 10 min with gentle agitation. The primary antibodies used were anti-Nab3 (mouse monoclonal 2F12) (Wilson *et al.* 1994) and anti-Sir2 (Garcia and Pillus 2002). Texas Red-conjugated goat anti-mouse and FITC-conjugated goat anti-rabbit were used as secondary antibodies. Staining was visualized with an Applied Precision Deltavision optical sectioning deconvolution microscope.

### Flow cytometry

Cell-cycle profiles were obtained by flow cytometry of propidium iodide stained cells on a FACSCalibur machine (Becton Dickinson) and analyzed with CellQuest software (Becton Dickinson). Cells were grown to an A_600_ of between 0.6 and 1.0, fixed in ethanol overnight, and stained with propidium iodide. Stained cells were sonicated and then analyzed by flow cytometer. For each sample, 100,000 cells were counted and analyzed.

## Results

### Four suppressors of the *esa1* temperature-sensitive phenotype

**Figure 1:**
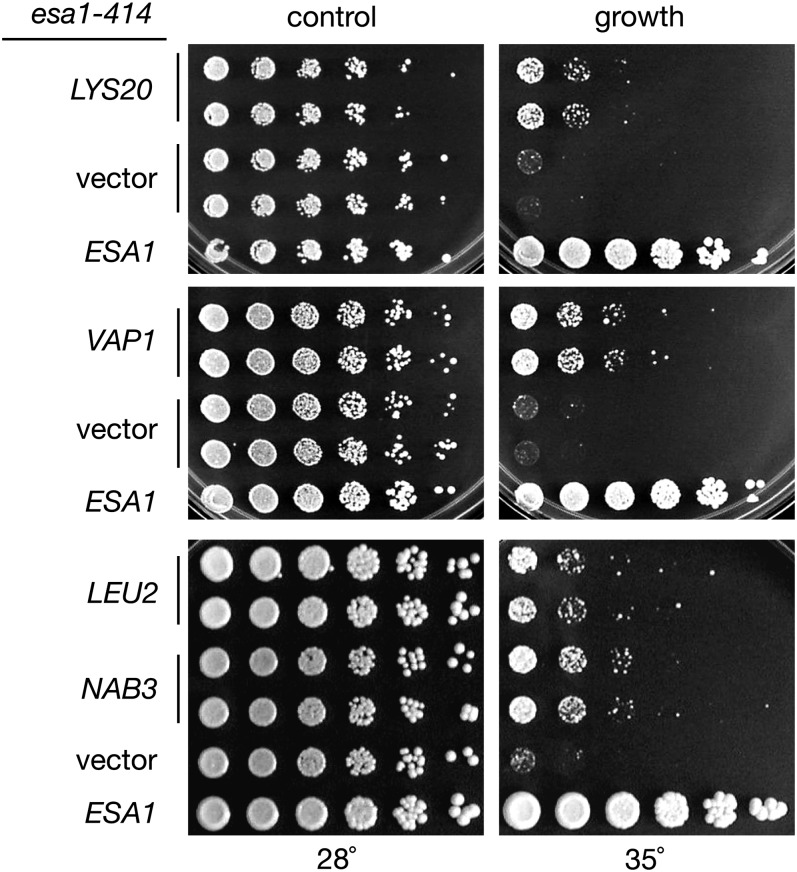
The *esa1* temperature-sensitive growth defect is partially suppressed by four genes expressed from 2μ plasmids. Increased gene dosage of *LYS20* (pLP1405), *VAP1* (pLP1406), *LEU2* (pLP1417), or *NAB3* (pLP1419) from a 2μ plasmid moderately suppresses the *esa1-414* (LPY3291) growth defect at 35°. The *esa1* growth defect at 35°, demonstrated by vector (pLP1402) transformants, is completely restored in cells transformed with an *ESA1* plasmid (pLP796). All strains were plated on SC-Ura-Trp media. Suppression was not observed at higher temperatures. Multiple independent transformants were tested to examine any variability between transformants.

To identify genes that interact functionally with *ESA1*, a dosage-suppressor screen was performed utilizing a genomic 2μ plasmid library. The *esa1-414* temperature-sensitive strain was transformed with the library, and transformants were tested for growth at both permissive and restrictive temperatures. Plasmids were rescued from transformants that grew at the restrictive temperature to determine the identity of suppressing genomic fragments. The results of this analysis revealed four *esa1* dosage suppressors: *LEU2*, *LYS20*, *NAB3*, and *VAP1* (Figure 1). None of these suppressors bypassed the inviable *esa1Δ*. When tested with other previously characterized *esa1* alleles (Clarke *et al.* 1999), some allele-specificity was observed (supporting information, Table S1). The series of alleles was also tested for suppression of other *esa1* phenotypes (see below).

*LEU2* and *LYS20* are nonessential genes required for the biosynthesis of leucine and lysine [reviewed in Kohlhaw (2003) and Xu *et al.* (2006), respectively]. *VAP1* is also involved in amino acid metabolism, encoding a transporter of several amino acids, including tyrosine, tryptophan, valine, and leucine (Schmidt *et al.* 1994). Characterization of *LYS20* as a suppressor of *esa1* revealed additional roles for this metabolic gene in DNA damage repair (Scott and Pillus 2010). *NAB3*, as noted, is an essential gene critical for 3′-end processing of nonpolyadenylated transcripts [reviewed in Lykke-Andersen and Jensen (2007)].

### Increased dosage of *NAB3* suppresses multiple *esa1* mutant phenotypes

**Figure 2:**
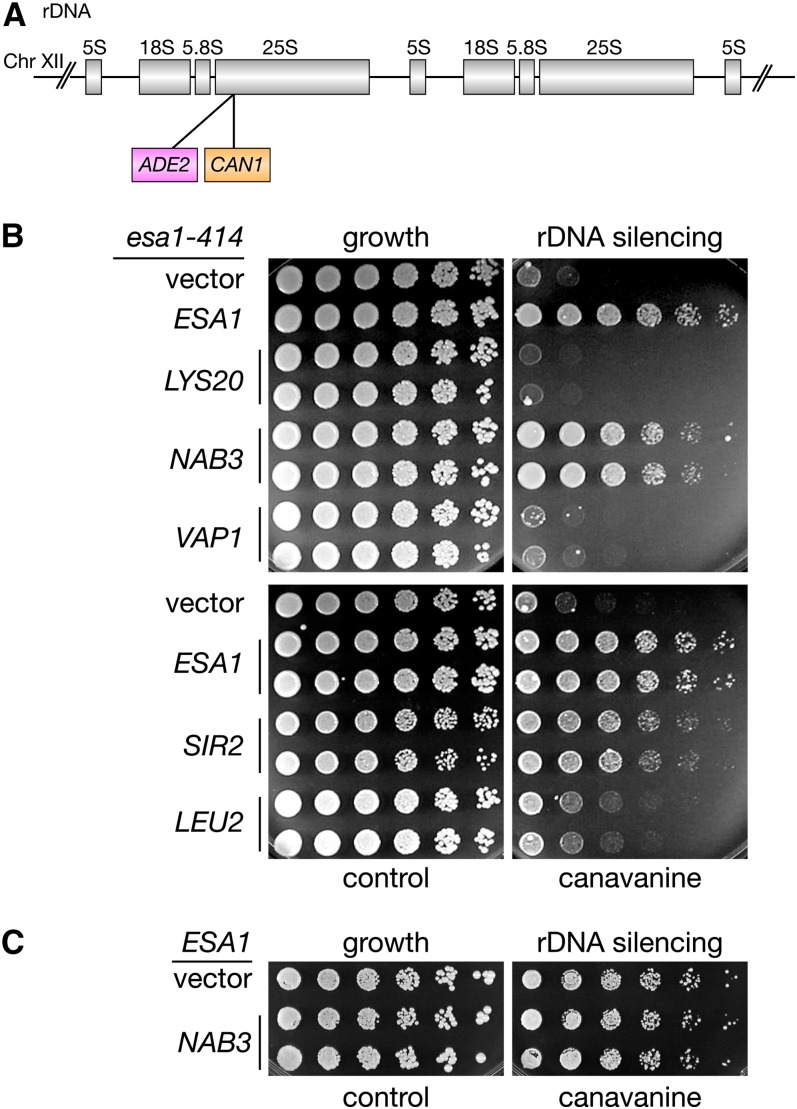
The *esa1* rDNA silencing defect is suppressed by increased gene dosage of *NAB3*. (A) Diagram and location of rDNA::*ADE2-CAN1* silencing marker within the rDNA array on chromosome XII. (B) The *esa1* rDNA silencing defect (vector) is restored in cells transformed with an *ESA1* plasmid, and by *NAB3*. Increased dosage of *LYS20*, *LEU2*, or *VAP1* does not rescue *esa1*’s rDNA silencing defect. An *esa1* strain with the 25S rDNA::*ADE2-CAN1* reporter (LPY4911) was transformed with vector (pLP1402), *ESA1* (pLP796), *LYS20* (pLP1412), *NAB3* (pLP1238), *VAP1* (pLP1259), *SIR2* (pLP37), or *LEU2* (pLP1417). To test for rDNA silencing defects, strains were plated on SC-Ade-Arg-Ura (growth) with and without 32 µg/ml canavanine (rDNA silencing) at 33°. (C) *NAB3* overexpression (pLP1238) has no effect on rDNA silencing of a WT strain (LPY4909).

To understand the connection between the suppressors and Esa1 function, overexpression of the four genes was tested for suppression of *esa1* mutant defects other than temperature sensitivity. One phenotype of *esa1* mutants is a strong rDNA-silencing defect and a slight increase in mitotic rDNA recombination (Clarke *et al.* 2006). Previously, it was shown that increased gene dosage of *SIR2* suppresses the *esa1* rDNA-silencing defect (Clarke *et al.* 2006). Using an *esa1* strain with the *ADE2-CAN1* dual reporter integrated at a single 25S rDNA repeat (Fritze *et al.* 1997) (Figure 2A), the suppressors were tested for their effect on silencing of the rDNA locus. Only increased dosage of *NAB3* robustly suppressed the *esa1* rDNA-silencing defect, restoring silencing to near wild-type levels (Figure 2B). By contrast, *LYS20* slightly exacerbated *esa1*’s silencing defect, whereas *LEU2* and *VAP1* had little to no effect (Figure 2B). Unlike increased gene dosage of *SIR2* in a wild-type strain (Smith *et al.* 1998b), *NAB3* did not enhance wild-type rDNA silencing (Figure 2C). None of the suppressors had significant effects on rDNA recombination. As there appeared to be a link between *NAB3* and *ESA1* for both silencing and growth, we chose to characterize *NAB3* in greater detail.

**Figure 3:**
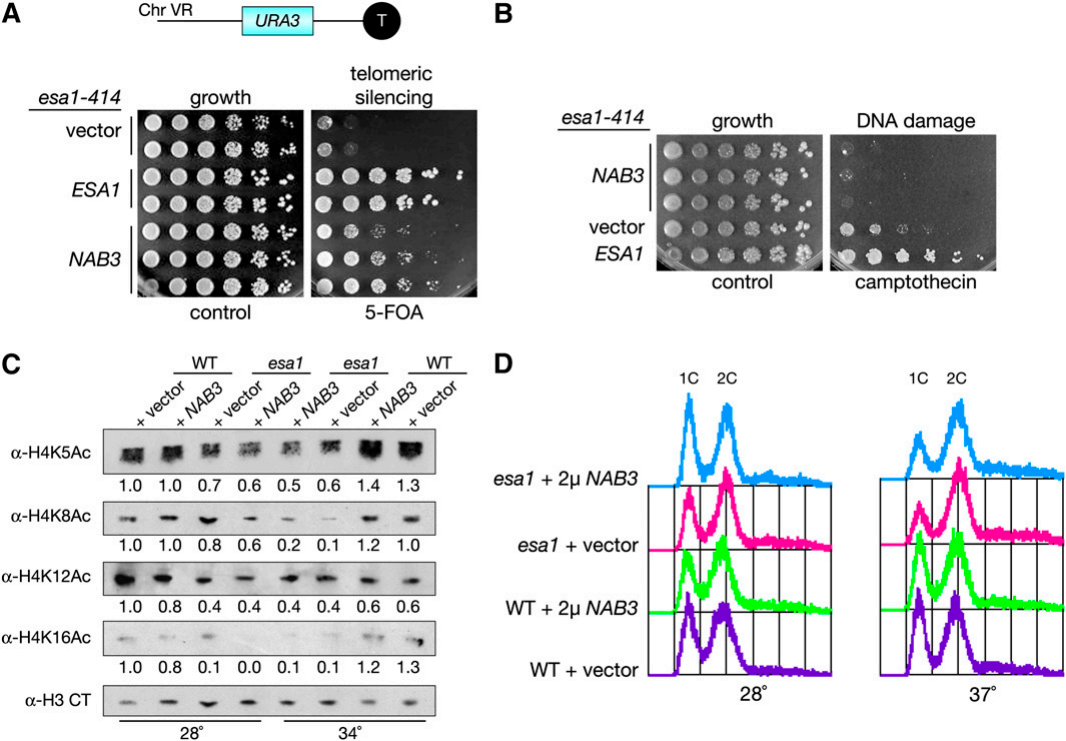
Overexpression of *NAB3* affects multiple *esa1* mutant phenotypes. (A) Top: Diagram of TELVR::*URA3* telomeric silencing marker on the right arm of chromosome V. Bottom: Increased gene dosage of *NAB3* suppresses the *esa1* 5-FOA sensitivity in this assay. An *esa1* strain with the TELVR::*URA3* reporter (LPY4919) was transformed with vector (pLP271), *ESA1* (pLP798), or *NAB3* (pLP1310), and plated on SC-Trp (growth) with and without 5-FOA (telomeric silencing) at 33°. (B) Increased gene dosage of *NAB3* exacerbates *esa1*’s sensitivity to the DNA damaging agent camptothecin. An *esa1* strain (LPY4774) was transformed with vector (pLP326), *ESA1* (pLP796), or *NAB3* (pLP2018), and plated on SC-Ura with DMSO (growth) and 20 µg/ml camptothecin (DNA damage). (C) Overexpression of *NAB3* does not increase global acetylation levels of H4K5, H4K8, H4K12, or H4K16 in *esa1* mutants. Whole-cell extracts were made from wild-type (LPY5) and *esa1* (LPY4774) strains with vector (pLP362) or 2μ *NAB3* (pLP2018) grown in SC-Ura media at both permissive (28°) and elevated (34°) temperatures. These were immunoblotted for amounts of isoform-specific H4 acetylation and total H3. An H3 reprobe was performed for each individual H4 acetylation blot. Quantification data shown are normalized for H3 loading. (D) Overexpression of *NAB3* does not influence *esa1*’s G2/M cell-cycle block. The same strains as in (C) were grown at 28° and shifted to 37° for 4 hr before fixing and staining with propidium iodide. Cell-cycle profiles were analyzed by flow cytometry.

In addition to their rDNA-silencing defects, *esa1* mutants are defective in telomeric silencing (Clarke *et al.* 2006) (Figure 3A), as shown by diminished growth on 5-FOA when using a *URA3* reporter gene on the right arm of chromosome V (TELVR) (Renauld *et al.* 1993). Increased dosage of *NAB3* in *esa1* mutants allowed for increased growth on 5-FOA, thereby rescuing the sensitivity shown in the *esa1* mutant (Figure 3A). Recent studies have shown that readout of this reporter-based assay for some genes may reflect changes in nucleotide metabolism instead of telomeric-silencing defects (Rossmann *et al.* 2011; Takahashi *et al.* 2011). Thus, based on these new studies, rescue of *esa1*’s 5-FOA sensitivity by *NAB3* in strains carrying the *URA3* telomeric reporter gene can be interpreted as the ability of *NAB3* overexpression to suppress telomeric-silencing defects or nucleotide metabolism changes in an *esa1* mutant. Because *ESA1* has no known defects in *HM* silencing or mating efficiency (Clarke *et al.* 2006), *NAB3* dosage was not tested for effects on mating efficiency.

Another phenotype of *esa1* mutants is sensitivity to DNA damage induced by camptothecin, a topoisomerase I inhibitor that triggers double-strand breaks (Bird *et al.* 2002). *NAB3* overexpression was tested for its ability to suppress this *esa1* mutant defect in the DNA damage response and was found to exacerbate *esa1*’s camptothecin sensitivity (Figure 3B). This result is in contrast to *NAB3*-mediated suppression of *esa1*’s silencing defects, highlighting a difference between Nab3 and Esa1’s functions in transcriptional silencing and DNA damage repair.

At a molecular level, global H4 acetylation is dramatically reduced in *esa1* mutants when grown at restrictive temperatures (Clarke *et al.* 1999). To determine whether increased dosage of *NAB3* restores wild-type levels of histone acetylation to *esa1* mutants, a series of protein immunoblots with isoform-specific antibodies was performed to define the global acetylation state in *esa1* strains overexpressing *NAB3*. All the histone H4 lysine residues that Esa1 is known to acetylate (K5, K8, K12, and K16) (Clarke *et al.* 1999) were tested in these experiments (Figure 3C). Total histone levels were determined by probing with a control antibody specific to the C-terminus of histone H3. This series of immunoblots shows that increased dosage of *NAB3* in *esa1* strains did not restore H4 acetylation. Therefore, *NAB3* overexpression does not rescue *esa1* mutants by restoring global acetylation defects at substrate residues in the H4 N-terminal tail.

A distinct potential mechanism for *NAB3* suppression is through Esa1’s role in the cell cycle. Since Esa1 is required for cell-cycle progression through G2/M, cell-cycle profiles of *esa1* mutant strains with increased dosage of *NAB3* were examined by flow cytometry to distinguish cellular DNA content before (1C) and after (2C) replication. The *esa1* mutants at restrictive temperature have a well-defined G2/M cell-cycle block, visualized as a decrease in the 1C peak and an accumulation of the 2C peak (Clarke *et al.* 1999). With *NAB3* overexpression, no change in the *esa1* cell-cycle profile was observed (Figure 3D), indicating that *NAB3* overexpression does not bypass the G2/M cell-cycle block of *esa1* mutants. Thus, increased dosage of *NAB3* suppresses a defined subset of *esa1* mutant phenotypes, which includes silencing defects and temperature sensitivity.

### Nab3 does not affect protein or transcript levels of histone-modifying enzymes

**Figure 4:**
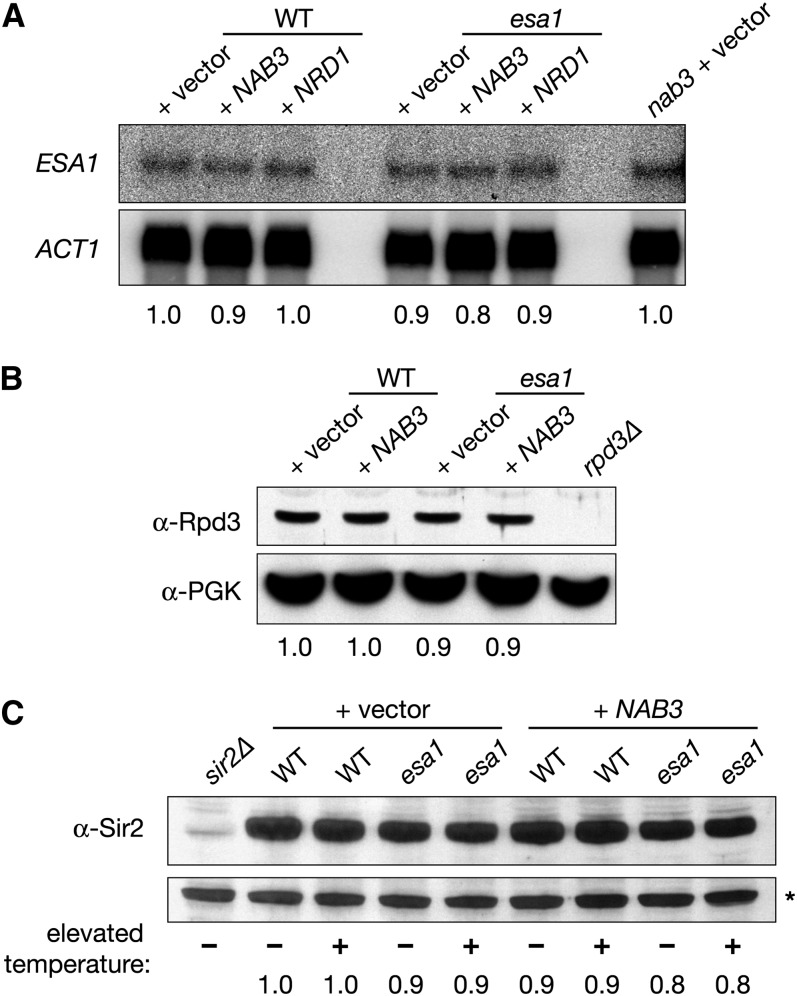
*NAB3* overexpression does not change transcript levels of *ESA1* or protein levels of Rpd3 and Sir2. (A) *NAB3* overexpression does not alter *ESA1* mRNA levels. Total RNA was isolated from both WT (LPY5) and *esa1* (LPY4774) mutant strains grown at an elevated temperature (35°) with vector control (pLP362), *NAB3* overexpression (pLP2018), or *NRD1* overexpression (pLP2054). Northern analysis was performed with an *ESA1*-specific probe, and results were obtained by phosphorimager scan. (B) Overexpression of *NAB3* does not influence Rpd3 protein levels. Whole-cell lysates from WT and *esa1* strains grown at an elevated temperature (35°) with vector control or *NAB3* overexpression [same strains as in (A)] were examined by immunoblot with anti-Rpd3. An *rpd3Δ* strain (LPY12154) transformed with vector (pLP362) was used as a negative control, and anti-PGK1 (phosphoglycerate kinase) was used to determine equal loading between samples. (C) Overexpression of *NAB3* does not influence Sir2 protein levels. Whole-cell lysates were made from WT (LPY5) and *esa1* (LPY4774) strains grown at an elevated temperature (37°) with vector control (pLP1402) or *NAB3* overexpression (pLP1238) and immunoblotted with anti-Sir2. Extract from a *sir2Δ* strain was used as a negative control. A nonspecific band (*) detected by anti-Sir2 was used to determine equal loading between samples.

In addition to their function for termination of noncoding RNAs, there is evidence that Nab3 and its partner Nrd1 participate in 3′-end formation of protein-coding transcripts (Sugimoto *et al.* 1995; Arigo *et al.* 2006a; Darby *et al.* 2012). We considered the possibility that Nab3 might bind to *ESA1* mRNA to direct its 3′-end formation. Nab3 binding sites have the simple UCUU consensus sequence (Carroll *et al.* 2004) that is found at several positions within the *ESA1* transcript. Northern blotting was performed to determine whether there were any *NAB3*-dependent changes in the *ESA1* transcript. *NAB3* is an essential gene (Wilson *et al.* 1994) and, thus, the temperature-sensitive *nab3-10* mutant was used in this study. The *nab3-10* allele was described previously and specifies a single F371L amino acid substitution in its RNA-recognition motif (RRM) domain (Conrad *et al.* 2000). When *ESA1* transcripts were examined in the *nab3-10* mutant (Figure 4A), there were no detectable changes in either transcript levels or migration. Transcript levels of *ESA1* were also found to be constant with or without increased dosage of *NAB3* (Figure 4A). Increased dosage of *NRD1*, which encodes a binding partner of Nab3, also failed to influence *ESA1* mRNA. In conclusion, *NAB3* does not affect the transcriptional regulation of *ESA1* itself.

We considered the possibility that *NAB3* affects transcription of a histone deacetylase (HDAC) that acts in opposition to Esa1. Transcriptional downregulation of an HDAC could compensate for the lack of functional Esa1 and restore the imbalance of acetylation in the cell. For example, deletion of the histone deacetylase gene *RPD3* suppresses the temperature-sensitivity and silencing defects of an *esa1* mutant (Chang and Pillus 2009). To test whether *NAB3* suppression of *esa1* is mediated through changes in Rpd3 levels, its protein levels were examined by immunoblot. Comparing Rpd3 levels between wild type and *esa1* strains with and without increased dosage of *NAB3* revealed no *NAB3*-dependent changes (Figure 4B). Another HDAC candidate of interest is Sir2, an HDAC critical for establishment and maintenance of silent chromatin [reviewed in Rusche *et al.* (2003)]. Similar to *NAB3* overexpression, *SIR2* overexpression has been shown to rescue rDNA silencing in an *esa1* mutant (Clarke *et al.* 2006). Sir2 levels were determined in wild-type and *esa1* mutant strains overexpressing *NAB3* by immunoblot, and no *NAB3*-dependent differences were observed (Figure 4C). Therefore, *NAB3* overexpression does not alter expression of either Rpd3 or Sir2, demonstrating that suppression is not mediated through transcriptional regulation of either HDAC.

### *nab3* mutants share phenotypes with *esa1* mutants

**Figure 5:**
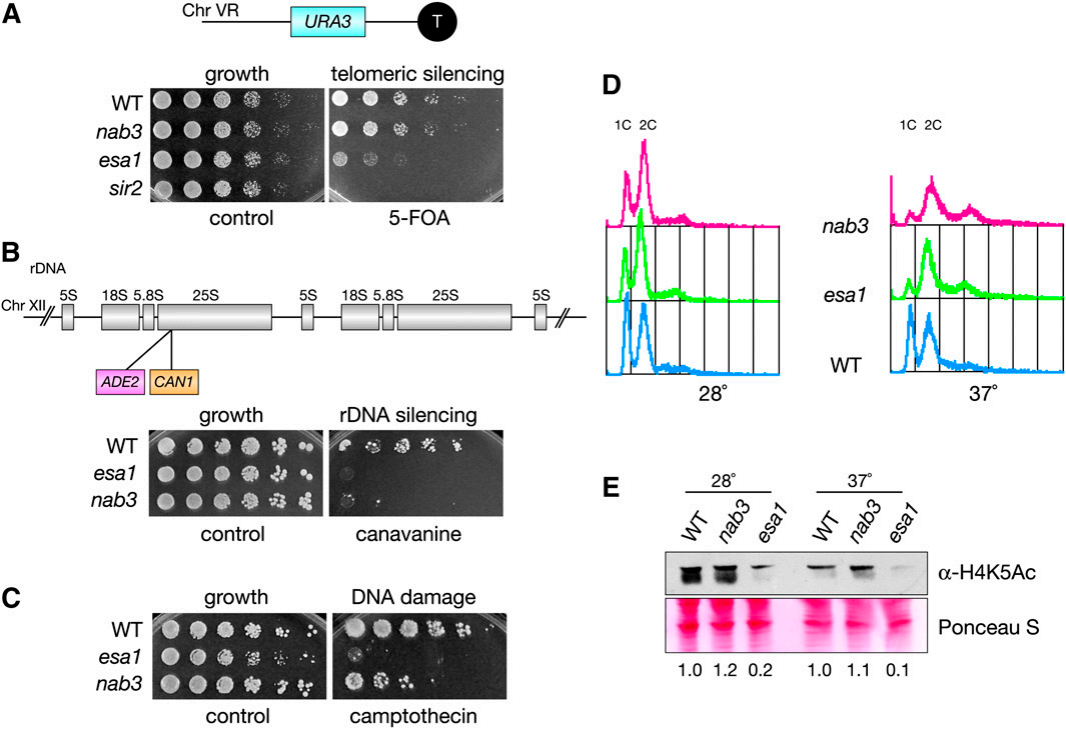
*nab3* mutants display defects similar to *esa1* mutants. (A) *nab3* mutants display no defect in the telomeric 5-FOA^S^ assay. WT (LPY4917), *nab3-10* (LPY5407), *esa1* (LPY4919), and *sir2* (LPY4979) strains with a TELVR::*URA3* reporter were plated on SC and 5-FOA. (B) *nab3* mutants have an rDNA silencing defect. WT (LPY4909), *esa1* (LPY4911), and *nab3* (LPY5406) strains with the 25S rDNA::*ADE2-CAN1* reporter were assayed for rDNA silencing defects on SC-Ade-Arg with and without 16 μg/ml canavanine. (C) The *nab3* mutant is sensitive to the DNA-damaging agent camptothecin. WT (LPY5), *esa1* (LPY4774), and *nab3* (LPY10622) were plated on DMSO (control) and camptothecin (40 μg/ml) to test for drug sensitivity. (D) *nab3* mutants display a G2/M block when grown at an elevated temperature. The same strains as in (C) were fixed and stained with propidium iodide to analyze cell-cycle profiles after being grown at 28° and shifted to 37° for 4 hr. (E) *nab3* mutants have wild-type levels of global H4K5 acetylation. The same strains as in (C) and (D) were grown in YPD at 28° and shifted to 37° for 2 hr before whole-cell extract preparation. Samples were immunoblotted to detect global H4K5 acetylation levels. Compared with the H4K5 acetylation levels in *esa1* mutants shown in Figure 3C, a more severe temperature challenge is shown here, accounting for the greater magnitude in decreased acetylation.

To characterize further the role of *NAB3* in relation to *ESA1*, *nab3* mutants were examined for established phenotypes of *esa1* mutants. Since *NAB3* overexpression rescued the *esa1* telomeric- and rDNA-silencing defects (Figures 2B and 3A), it was possible that *nab3* mutants might be defective in silencing. Telomeric silencing was tested using the same *URA3* reporter assay as before (Figure 3A), and it revealed that *nab3* mutants display growth on 5-FOA comparable to wild-type strains (Figure 5A). Use of an independent *ADE2* color-based telomeric-silencing assay also showed no defects for *nab3* mutants (Figure S1). Combined with the lack of defects observed for *nab3* mutants in both our assays (Figure 5A), the earlier observation that *NAB3* overexpression rescued *esa1*’s 5-FOA sensitivity (Figure 3A) likely results through an indirect mechanism. In contrast, when assayed for rDNA-silencing defects, *nab3* mutants displayed a strong defect, similar to that observed in *esa1* (Figure 5B). Together, these data suggest that Nab3 functions directly in rDNA silencing but not telomeric silencing.

*NAB3* overexpression did not suppress the DNA damage and cell-cycle phenotypes of *esa1* mutants (Figure 3, B and D). However, when *nab3* mutants were examined for defects in DNA damage repair and cell-cycle progression, the results revealed a role for *NAB3* in these processes. As seen in Figure 5C, *nab3* mutants are sensitive to the topoisomerase I inhibitor camptothecin, although less so than *esa1*. Cell-cycle profiles of *nab3* mutants also showed a G2/M block resembling that of *esa1* mutants (Figure 5D). In addition to the defective rDNA silencing of *nab3*, the identification of these phenotypes for *nab3* mutants reveals a more extensive functional overlap with *esa1* mutants.

We earlier considered the possibility that a molecular link for Nab3 and Esa1 functions would be that Nab3 influences histone acetylation (Figure 3C). When tested for changes in acetylation of H4K5, the primary *in vivo* target of Esa1 (Clarke *et al.* 1999), global acetylation in *nab3* mutants was maintained at wild-type levels (Figure 5E). Therefore, *NAB3* does not directly influence the global histone acetylation activity of Esa1’s primary target.

### Localization and posttranslational acetylation of Nab3 are altered in *esa1* mutants

The nucleolus is a key compartment for RNA processing in the nucleus. Ultrastructural analysis has shown *esa1* mutants to have aberrant nucleoli (Clarke *et al.* 1999), and *esa1* mutants display strong rDNA-silencing defects and rDNA chromatin structure defects (Clarke *et al.* 2006). Because of these connections of Esa1 to nucleolar function and Nab3′s influence on rDNA silencing (Figure 5B), Nab3 localization was visualized in *esa1* mutants. Immunofluorescence was performed using an antibody directed against Nab3 in wild-type and *esa1* strains. In addition, Sir2 staining was used to demarcate the nucleolus.

**Figure 6:**
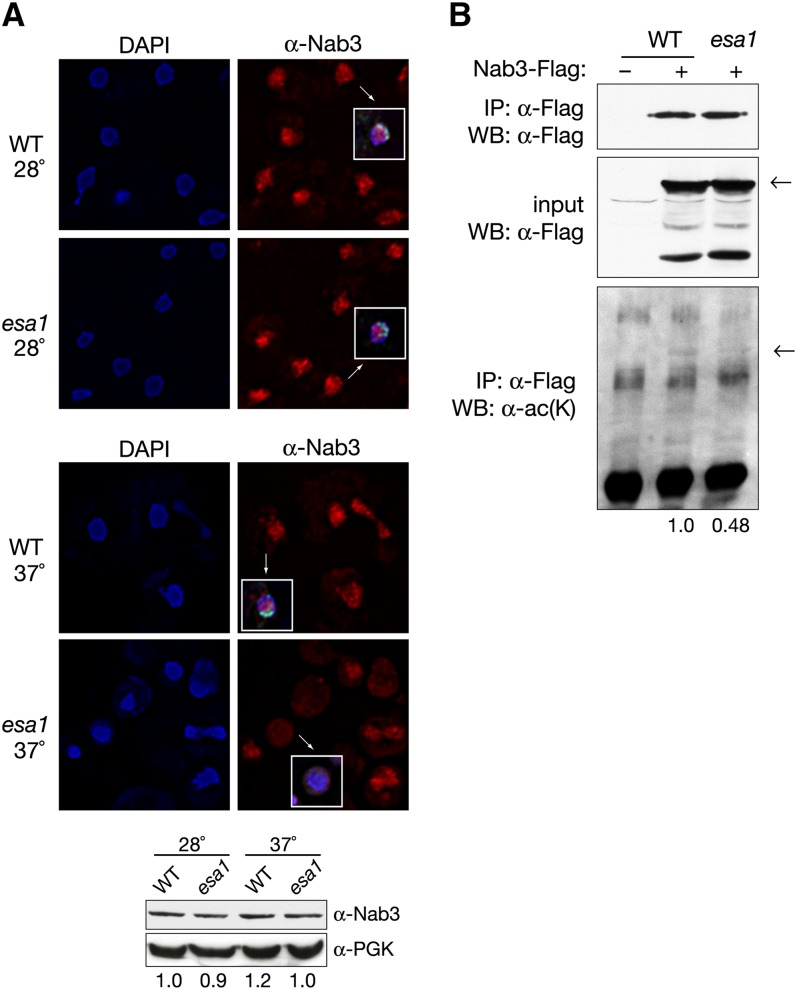
Nab3 localization and acetylation is altered in *esa1* cells. (A) Nab3 localization is aberrant in the *esa1* mutant. Top: At a permissive temperature (28°), Nab3 staining in wild type (LPY4909) and *esa1* (LPY4911) cells appears as punctate nuclear foci interspersed with diffuse nuclear staining. Sir2 localization demarcates the nucleolus in a crescent shape (inset, green) and is normal. At a restrictive temperature (37°), Nab3 staining is diffuse in the *esa1* mutant but appears normal in the wild-type strain. No Sir2 foci are observed in the *esa1* mutant. Bottom: WT and *esa1* strains used above were grown at permissive and elevated temperatures and used for immunoblots to detect total Nab3 levels using anti-Nab3. Anti-PGK1 (phosphoglycerate kinase) was used as a loading control. (B) Nab3 is acetylated *in vivo* in an *ESA1*-dependent manner. To detect posttranslational acetylation of Nab3, a WT (LPY15000) and *esa1* (LPY15004) strain containing a chromosomal FLAG-tagged version of Nab3 were grown at an elevated temperature (37°) and used in an anti-FLAG immunoprecipitation followed by an immunoblot with anti-acetyl lysine. Decreased levels of Nab3 acetylation are observed in the *esa1* mutant. Quantification of films from independent experiments shows a 48% decrease in Nab3 acetylation in *esa1* compared with wild-type. An untagged WT strain (LPY5) is used as a negative control. Nab3-FLAG levels are not themselves altered in the *esa1* mutant, as demonstrated by control immunoblotting of immunoprecipitations and inputs with anti-FLAG.

Nab3 localization has been previously described as dispersed throughout the nucleus but distinct from nucleolar structure proteins (Wilson *et al.* 1994) (Figure 6A, top). At permissive temperatures, Nab3 localization appeared normal in both wild-type and *esa1* cells. However, at restrictive temperature, Nab3 localization in *esa1* became diffuse and no longer confined to the nucleus as defined by DAPI staining (Figure 6A, middle), indicating that Nab3 localization is altered in the *esa1* mutant. Sir2 staining was also affected in the *esa1* mutant and no longer found in discrete nucleolar and telomeric foci, although Sir2 protein expression appeared essentially normal at elevated temperature (Figure 4C). Nab3 protein levels were also found to be equal by immunoblot between wild-type and *esa1* cells at both permissive and restrictive temperatures (Figure 6A, bottom).

Because WT levels of Nab3 were observed in *esa1* mutants, whereas simple overexpression suppressed *esa1* phenotypes, we considered the possibility that in the mutants, Nab3 protein differs not quantitatively but qualitatively. One such qualitative difference could be at the level of its posttranslational modification. We tested the idea that Nab3 might itself be an *in vivo* substrate for Esa1, a possibility first raised by a proteomics survey suggesting that Esa1 could acetylate Nab3 *in vitro* (Lin *et al.* 2009). To examine whether this modification occurs *in vivo*, an antibody that recognizes proteins with acetylated lysines was utilized. Immunoprecipitation of Nab3 followed by immunoblot detection with anti-acetyl-lysine revealed Nab3 to be acetylated *in vivo* (Figure 6B). To test whether Nab3 is a substrate for Esa1 acetylation, Nab3 acetylation levels were evaluated in an *esa1* mutant. Since *ESA1* is essential, the temperature-sensitive *esa1* mutant was grown at nonpermissive temperatures and samples were prepared. As expected if Esa1 acetylates Nab3 *in vivo*, a decrease in acetylated-Nab3 was observed in the *esa1* mutant. Quantification of the anti-acetyl-lysine immunoblot shows that Nab3 acetylation was reduced in the *esa1* mutant to 48% of the level observed in the wild-type strain. Thus, it appears that a fraction of the acetylation of Nab3 was *ESA1*-dependent, although our data did not distinguish whether this acetylation was by Esa1 on Nab3 directly as a target or indirectly through another acetyltransferase influenced by Esa1.

## Discussion

Genetic suppression has provided a valuable tool for expanding the understanding of Esa1’s nuclear functions (Biswas *et al.* 2008; Lin *et al.* 2008; Chang and Pillus 2009; Scott and Pillus 2010) and, in this case, its interactions with the RNA binding protein Nab3. Increased dosage of *NAB3* was found to suppress a subset of *esa1* mutant phenotypes, including temperature sensitivity and silencing defects. In addition, *nab3* mutants shared overlapping phenotypes with *esa1* mutants, displaying defects in rDNA silencing, cell-cycle progression, and the DNA damage response. Further strengthening these genetic interactions, nuclear localization and posttranslational acetylation of Nab3 were both altered in the *esa1* mutant.

Nab3 is found in a complex with the RNA binding protein Nrd1 and the Sen1 helicase. This Nab3 complex ensures proper termination and 3′-end formation of many nonpolyadenylated transcripts, including snRNAs, snoRNAs, and CUTs (Steinmetz *et al.* 2001; Arigo *et al.* 2006b; Thiebaut *et al.* 2006). In addition, Nab3 physically associates with the nuclear exosome for processing and degradation of these transcripts (Vasiljeva and Buratowski 2006). Nab3 and Nrd1 form a heterodimer (Carroll *et al.* 2007), and each protein has a different consensus RNA recognition sequence (Carroll *et al.* 2004). Domain analysis suggests that both proteins bind RNA transcripts, whereas Nrd1 also physically associates with the C-terminal domain of Pol II (Conrad *et al.* 2000). In accordance with these tightly linked functions of Nab3 and Nrd1, we found that overexpression of *NRD1* also suppresses some *esa1* mutant phenotypes (Figure S2). Because genetic suppression by *NRD1* was less dramatic than that by *NAB3*, our focus in this study was on *NAB3*’s genetic interaction with *ESA1*, but our observations with *NRD1* support the idea that suppression is mediated by Nab3 in the context of the Nab3-Nrd1-Sen1 complex, and not via an independent role of Nab3 alone.

Because the *ESA1* transcript was unchanged in the *nab3-10* mutant (Figure 4A), this implies that the Nab3-Nrd1-Sen1 complex does not direct 3′-end termination of the *ESA1* transcript. It should be noted that our study was restricted to this loss-of-function *nab3-10* mutation. Thus, considering the genetic limitations of studying essential genes such as *NAB3*, we cannot fully eliminate the possibility that the Nab3 complex processes the *ESA1* transcript, as we have not studied multiple mutant alleles of *NAB3*. However, we consider our *in vivo* data showing that Nab3 acetylation is influenced by Esa1 either directly or indirectly (Figure 6B) to provide a more likely explanation for the dosage suppression observed between *ESA1* and *NAB3*. Consistent with these data, one potential model for the suppression is that Esa1 acetylation of Nab3 influences its function such that the reduced Nab3 acetylation in *esa1* mutants results in its reduced cell viability and defects in rDNA silencing. Thus, suppression of these defects is obtained in the *esa1* mutant by overexpressing *NAB3* to compensate for the decreased pools of acetylated Nab3.

In *S. cerevisiae*, the rDNA is a repetitive array in the genome that is mainly transcribed by Pol I and Pol III. Reporter genes that are transcribed by Pol II and inserted in the array are known to undergo Sir2-mediated transcriptional silencing. An endogenous Pol II transcript has been detected in the “nontranscribed” spacer region (NTS1) of the rDNA. This transcript is a CUT that is processed by the Nab3 complex and degraded by the exosome (Houseley *et al.* 2007; Vasiljeva *et al.* 2008). In addition to uncovering an rDNA-silencing defect for *nab3* mutants (Figure 5B), we observed that overexpression of *NAB3* rescued the rDNA-silencing defects of *esa1* mutants (Figure 2). Esa1 binding is enriched at the rDNA, and histone acetylation at the rDNA is reduced in the *esa1* mutant (Clarke *et al.* 2006). Although Nab3 does not appear nucleolar by immunofluorescence (Wilson *et al.* 1994) (Figure 6A), a recent study found that Nab3 localizes to the rDNA via chromatin immunoprecipitation (Lepore and Lafontaine 2011). Thus, one possibility is that Nab3 recruitment to the CUTs within the rDNA is regulated by its acetylation status through Esa1 activity. Future studies will establish how Esa1 functions with the Nab3-Nrd1 complex in contributing to transcriptional silencing at the rDNA.

The number of nonhistone proteins known to be acetylated by Esa1 and the MYST family of KATs has expanded in recent years. Several schools of thought exist about the function of this posttranslational modification. In parallel with the models for histone acetylation, acetylation of nonhistone proteins may change the activity of these proteins or may serve as a recruitment platform for physical binding of other proteins [reviewed in Sapountzi and Côté (2011)]. Our finding that Nab3 is acetylated *in vivo* raises several possibilities regarding the function of this posttranslational modification. Whereas Nab3 acetylation is reduced in an *esa1* mutant, overall levels of Nab3 remain constant (Figure 6). Therefore, it is unlikely that acetylation affects Nab3 stability but, rather, that it influences its activity or function. Knowing that Nab3 is aberrantly localized in the *esa1* mutant, one possible scenario is that acetylation of Nab3 by Esa1 promotes proper Nab3 nuclear localization.

In contrast to *NAB3*, the other three suppressors identified in our dosage-suppression screen (*LEU2*, *LYS20*, and *VAP1*) are all involved in amino acid metabolism. A separate study defined the connections between *LYS20* and *ESA1* through DNA repair that could be distinguished from Lys20’s role in amino acid biosynthesis, potentially through a noncanonical role in acetylation (Scott and Pillus 2010). Recent findings report the prevalence of lysine acetylation as a posttranslational modification in the regulation of metabolic proteins in mammals (Zhao *et al.* 2010). In light of these studies and ours, it is possible that Esa1 acetylates the protein products of the genes we identified as dosage suppressors. Only Nab3, and not the other suppressors, was identified as a substrate in the *in vitro* proteomics study (Lin *et al.* 2009). However, a number of other metabolic enzymes were found, including the gluconeogenic enzyme Pck1 that is reciprocally deacetylated by Sir2, providing a link to our earlier suppression studies between *ESA1* and *SIR2* (Clarke *et al.* 2006). One potential explanation for our current findings of dosage suppression of *esa1* by *LEU2* and *VAP1* is that Leu2 and Vap1 are acetylated by Esa1 *in vivo*. Future studies to determine *in vivo* Esa1 targets of nonhistone proteins will shed light on additional substrates and their functions.

Although it has been assumed that Esa1’s catalytic activity is its essential activity, it is unclear exactly why *esa1Δ* strains are inviable. One recent study found that an *ESA1* strain bearing a mutation in a residue important for catalysis retained viability, proposing that there may be more to the essential nature of Esa1 than its histone acetyltransferase activity (Decker *et al.* 2008). Given that our screen highlights a strong genetic interaction between *ESA1* and the essential gene *NAB3*, along with several genes encoding metabolic proteins (*LEU2*, *VAP1*, *LYS20*), one of Esa1’s essential functions may be the recognition and acetylation of important nonhistone substrates.

Suppressor analysis is a widely used strategy that facilitates the identification of functional relationships between different proteins. A recent investigation of hundreds of dosage suppressors in yeast revealed that dosage suppression provides functional links between two genes (Magtanong *et al.* 2011). In addition, dosage suppression can identify unique interactions that are not discovered through other types of genome-wide studies, such as protein-protein and synthetic sickness interactions. In our study, genetic suppression has provided an effective platform for identifying and characterizing potential new substrates for an enzyme primarily studied as an acetyltransferase targeting histones.

## Supplementary Material

Supporting Information
