# Supplementary material for: Suppression Analysis of esa1 Mutants in Saccharomyces cerevisiae Links NAB3 to Transcriptional Silencing and Nucleolar Functions
Source: G3 (Bethesda). 2012 Oct 1;2(10):1223–32. doi: 10.1534/g3.112.003558 (PMC3464115; doi:10.1534/g3.112.003558)
Supplement: Supporting Information [file supp_2_10_1223__index.html]

Supporting Information 

# Suppression Analysis of *esa1* Mutants in *Saccharomyces cerevisiae* Links *NAB3* to Transcriptional Silencing and Nucleolar Functions

## Supporting Information for Chang, Clarke, and Pillus, 2012

**Files in this Data Supplement:**

- Supporting Information - Figures S1 and S2 and Table S1 (PDF, 2.1 MB)
- Figure S1 - Mutants of *nab3* do not display a telomeric silencing defect with an *ADE2* reporter at the left arm of chromosome VII (PDF, 989 KB)
- Figure S2 - Overexpression of *NRD1* suppresses esa1 phenotypes (PDF, 1.1 MB)
- Table S1 - The allele-specific suppression of *esa1* phenotypes (PDF, 76 KB)
