# Supplementary material for: Suppression Analysis of esa1 Mutants in Saccharomyces cerevisiae Links NAB3 to Transcriptional Silencing and Nucleolar Functions
Source: G3 (Bethesda). 2012 Oct 1;2(10):1223–32. doi: 10.1534/g3.112.003558 (PMC3464115; doi:10.1534/g3.112.003558)
Supplement: Supporting Information [file supp_2.10.1223_003558SI.pdf]

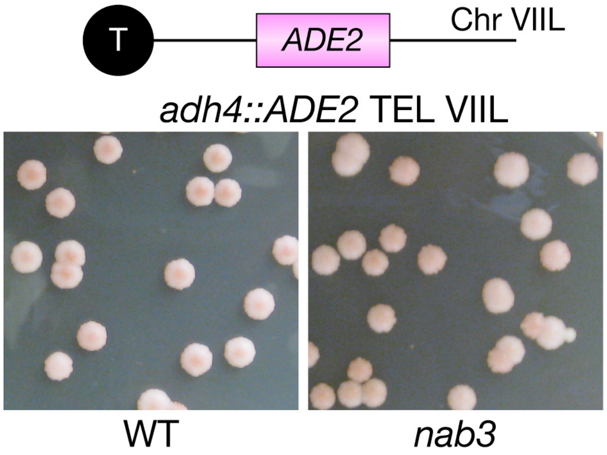

Figure S1 Mutants of *nab3* do not display a telomeric silencing defect with an *ADE2* reporter at the left arm of chromosome VII. WT (LPY11300) and *nab3* (LPY11286) strains with the *adh4::ADE2* TEL VIIL reporter were grown up overnight in YPD liquid cultures, and plated for single colonies on YPD. After 3 days growth at 30˚, plates were incubated for one month at 4˚ for pink color development before images were captured.

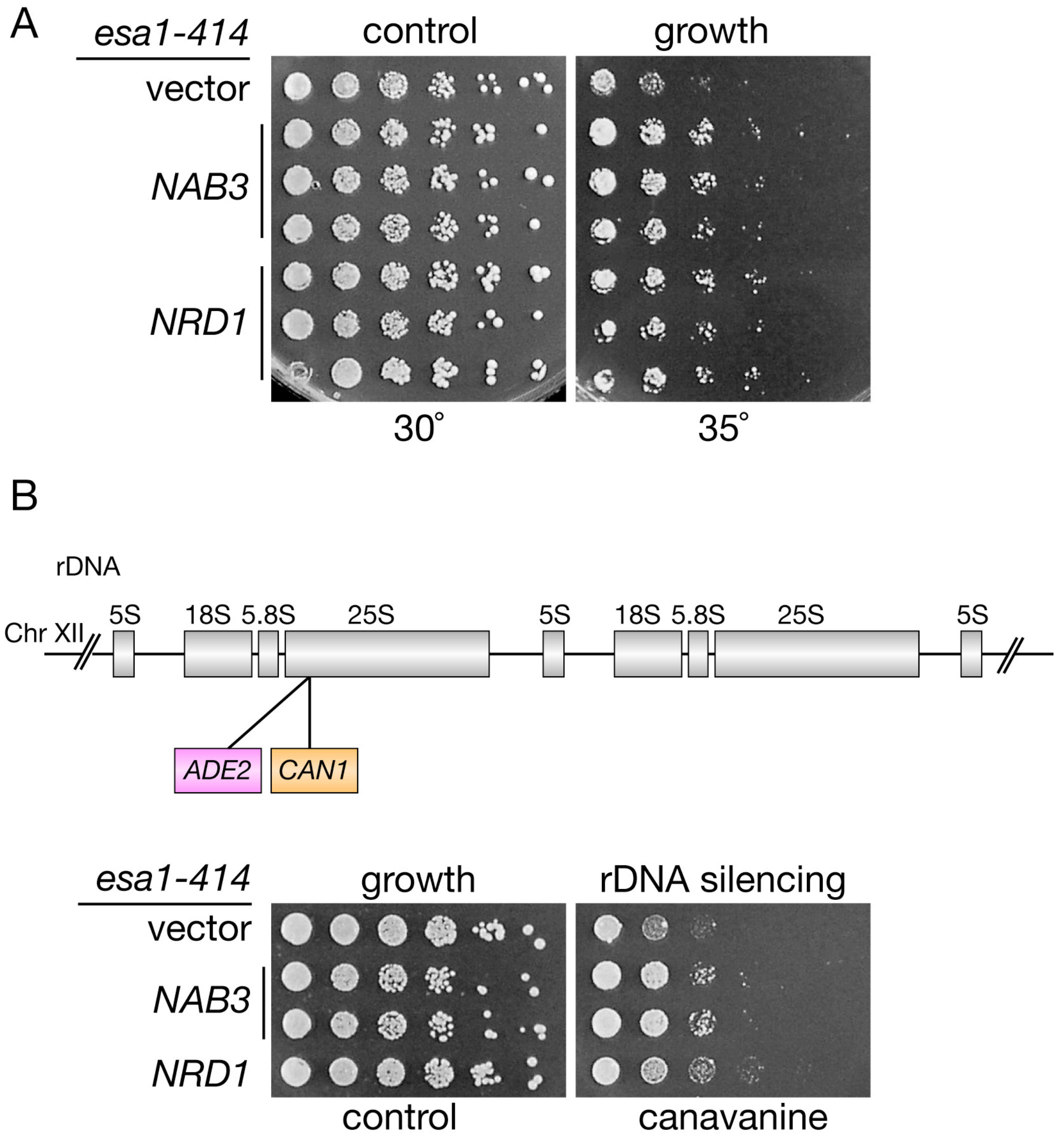

Figure S2 Overexpression of *NRD1* suppresses *esa1* phenotypes. A) Overexpression of *NRD1* suppresses the temperature-sensitivity of an *esa1* mutant. The *esa1* strain (LPY3291) was transformed with vector (pLP362), *NAB3* (pLP2018), or *NRD1* (pLP2054) 2-micron plasmids. Transformants were plated on SC-Ura-Trp at 30˚ for growth and 35˚ to observe suppression. B) Overexpression of *NRD1* suppresses the rDNA silencing defect of an *esa1* mutant. An *esa1* strain with the rDNA::*ADE2-CAN1* reporter was transformed with vector (pLP362), *NAB3* (pLP2018), or *NRD1* (pLP2054) 2-micron plasmids. Transformants were plated on SC-Ade-Arg-Ura with and without 32 µg/ml canavanine to assay for rDNA silencing.

Table S1 The allele-specific suppression of *esa1* phenotypes

Temperature-sensitive suppression

allele	*NAB3*	*LYS20*	*LEU2*	*VAP1*
esa1-414	+++	++	++	++++
esa1-L254P	–/+	+	++	+
esa1-L327S	–/+	–/+	–/+	++

rDNA silencing suppression

allele	*NAB3*	*LYS20*	*LEU2*	*VAP1*
esa1-414	+++	–	–/+	–/+
esa1-L254P	+++	–	not tested	not tested

+ indicates suppression of *esa1* phenotype (+++ signifies stronger suppression)

–/+ indicates no change in *esa1* phenotype

– indicates exacerbation of *esa1* phenotype
