# Supplementary material for: Suppression Analysis of esa1 Mutants in Saccharomyces cerevisiae Links NAB3 to Transcriptional Silencing and Nucleolar Functions
Source: G3 (Bethesda). 2012 Oct 1;2(10):1223–32. doi: 10.1534/g3.112.003558 (PMC3464115; doi:10.1534/g3.112.003558)
Supplement: Supporting Information [file supp_2.10.1223_FigureS1.pdf]

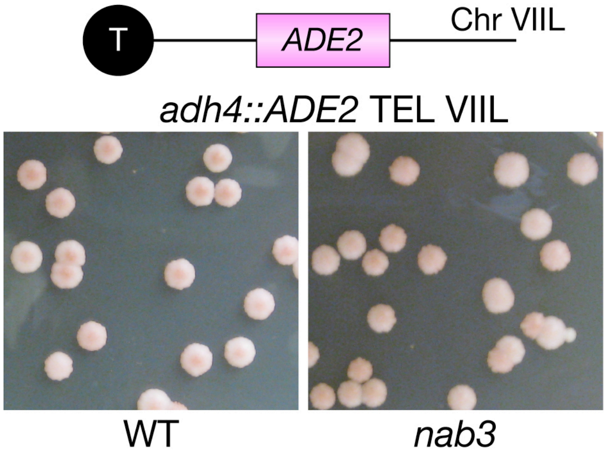

Figure S1 Mutants of *nab3* do not display a telomeric silencing defect with an *ADE2* reporter at the left arm of chromosome VII. WT (LPY11300) and *nab3* (LPY11286) strains with the *adh4::ADE2* TEL VIIL reporter were grown up overnight in YPD liquid cultures, and plated for single colonies on YPD. After 3 days growth at 30˚, plates were incubated for one month at 4˚ for pink color development before images were captured.
