# Supplementary material for: Suppression Analysis of esa1 Mutants in Saccharomyces cerevisiae Links NAB3 to Transcriptional Silencing and Nucleolar Functions
Source: G3 (Bethesda). 2012 Oct 1;2(10):1223–32. doi: 10.1534/g3.112.003558 (PMC3464115; doi:10.1534/g3.112.003558)
Supplement: Supporting Information [file supp_2.10.1223_FigureS2.pdf]

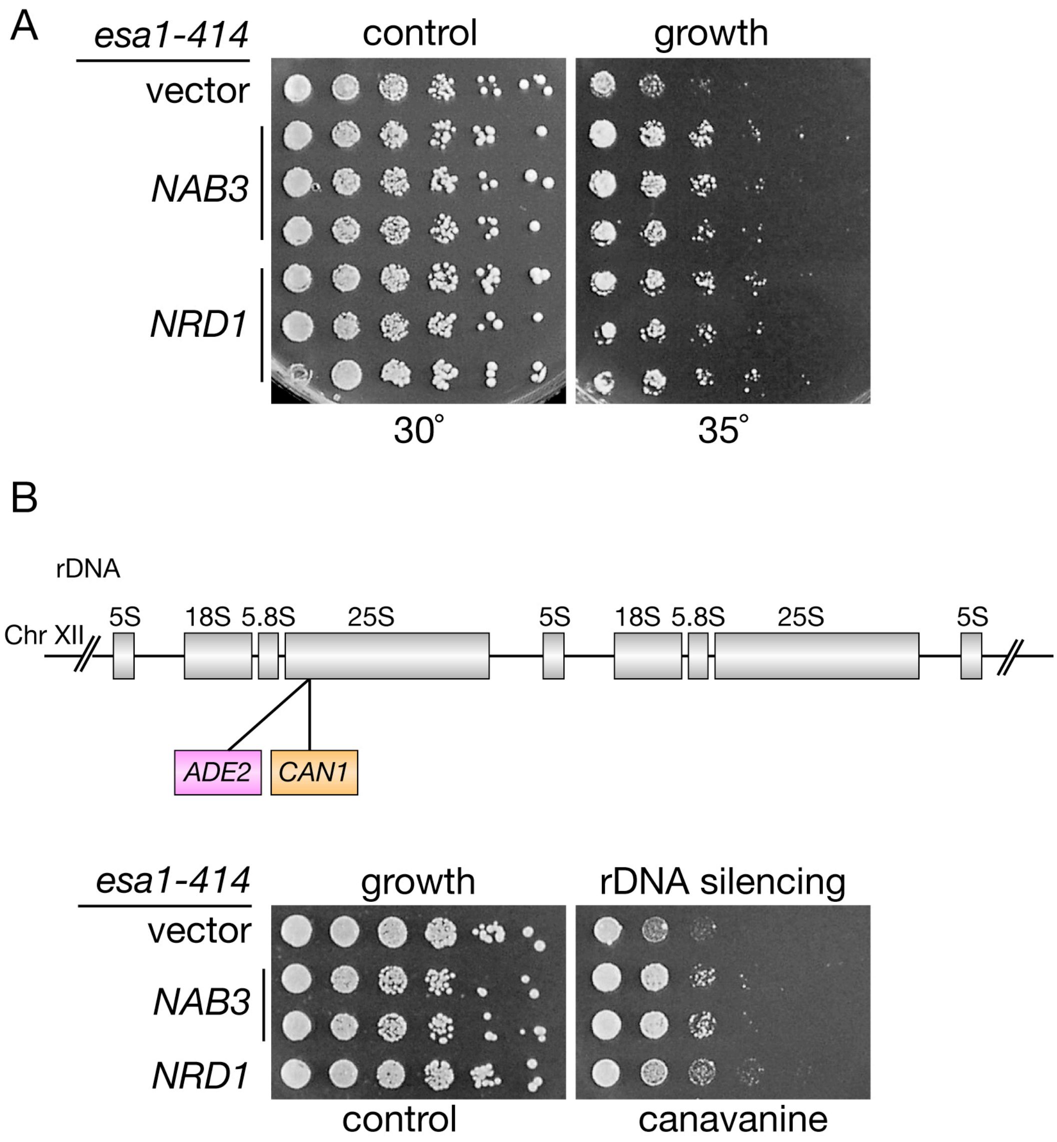

Figure S2 Overexpression of *NRD1* suppresses *esa1* phenotypes. A) Overexpression of *NRD1* suppresses the temperature-sensitivity of an *esa1* mutant. The *esa1* strain (LPY3291) was transformed with vector (pLP362), *NAB3* (pLP2018), or *NRD1* (pLP2054) 2-micron plasmids. Transformants were plated on SC-Ura-Trp at 30˚ for growth and 35˚ to observe suppression. B) Overexpression of *NRD1* suppresses the rDNA silencing defect of an *esa1* mutant. An *esa1* strain with the rDNA::*ADE2-CAN1* reporter was transformed with vector (pLP362), *NAB3* (pLP2018), or *NRD1* (pLP2054) 2-micron plasmids. Transformants were plated on SC-Ade-Arg-Ura with and without 32 µg/ml canavanine to assay for rDNA silencing.
