# Supplementary material for: Suppression Analysis of esa1 Mutants in Saccharomyces cerevisiae Links NAB3 to Transcriptional Silencing and Nucleolar Functions
Source: G3 (Bethesda). 2012 Oct 1;2(10):1223–32. doi: 10.1534/g3.112.003558 (PMC3464115; doi:10.1534/g3.112.003558)
Supplement: Supporting Information [file supp_2.10.1223_TableS1.pdf]

Table S1 The allele-specific suppression of *esa1* phenotypes

Temperature-sensitive suppression

allele	*NAB3*	*LYS20*	*LEU2*	*VAP1*
esa1-414	+++	++	++	++++
esa1-L254P	–/+	+	++	+
esa1-L327S	–/+	–/+	–/+	++

rDNA silencing suppression

allele	*NAB3*	*LYS20*	*LEU2*	*VAP1*
esa1-414	+++	–	–/+	–/+
esa1-L254P	+++	–	not tested	not tested

+ indicates suppression of *esa1* phenotype (+++ signifies stronger suppression)
–/+ indicates no change in *esa1* phenotype
– indicates exacerbation of *esa1* phenotype
